# Storytelling of Hypertrophic Cardiomyopathy Discovery

**DOI:** 10.3390/jcdd11100300

**Published:** 2024-09-28

**Authors:** Gaetano Thiene, Chiara Calore, Monica De Gaspari, Cristina Basso

**Affiliations:** Department of Cardiac, Thoracic, Vascular Sciences and Public Health, University of Padua Medical School, 35121 Padova, Italy; chiara.calore@unipd.it (C.C.); monica.deg1@gmail.com (M.D.G.); cristina.basso@unipd.it (C.B.)

**Keywords:** cardiomyopathies, history of medicine, hypertrophic cardiomyopathy, pathology, restrictive cardiomyopathy, sudden cardiac death, transplantation

## Abstract

The discovery of hypertrophic cardiomyopathy (HCM) dates back to 1958, when the pathologist Donald Teare of the St. George’s Hospital in London performed autopsies in eight cases with asymmetric hypertrophy of the ventricular septum and bizarre disorganization (disarray) at histology, first interpreted as hamartoma. Seven had died suddenly. The cardiac specimens were cut along the long axis, similar to the 2D echo. In the same year, at the National Institute of Health U.S.A., Eugene Braunwald, a hemodynamist, and Andrew Glenn Morrow, a cardiac surgeon, clinically faced a patient with an apparently similar morbid entity, with a systolic murmur and subaortic valve gradient. “Discrete” subaortic stenosis was postulated. However, at surgery, Dr. Morrow observed only hypertrophy and performed myectomy to relieve the obstruction. This first Braunwald–Morrow patient underwent a successful cardiac transplant later at the disease end stage. The same Dr. Morrow was found to be affected by the familial HCM and died suddenly in 1992. The term “functional subaortic stenosis” was used in 1959 and “idiopathic hypertrophic subaortic stenosis” in 1960. Years before, in 1957, Lord Brock, a cardiac surgeon at the Guy’s Hospital in London, during alleged aortic valve surgery in extracorporeal circulation, did not find any valvular or discrete subaortic stenoses. In 1980, John F. Goodwin of the Westminster Hospital in London, the head of an international WHO committee, put forward the first classification of heart muscle diseases, introducing the term cardiomyopathy (dilated, hypertrophic, and endomyocardial restrictive). In 1995, the WHO classification was revisited, with the addition of two new entities, namely arrhythmogenic and purely myocardial restrictive, the latter a paradox of a small heart accounting for severe congestive heart failure by ventricular diastolic impairment. A familial occurrence was noticed earlier in HCM and published by Teare and Goodwin in 1960. In 1989–1990, the same family underwent molecular genetics investigation by the Seidman team in Boston, and a missense mutation of the β-cardiac myosin heavy chain in chromosome 14 was found. Thus, 21 years elapsed from HCM gross discovery to molecular discoveries. The same original family was the source of both the gross and genetic explanations of HCM, which is now named sarcomere disease. Restrictive cardiomyopathy, characterized grossly without hypertrophy and histologically by myocardial disarray, was found to also have a sarcomeric genetic mutation, labeled “HCM without hypertrophy”. Sarcomere missense mutations have also been reported in dilated cardiomyopathy (DCM) and non-compaction cardiomyopathy. Moreover, sarcomeric gene defects have been detected in some DNA non-coding regions of HCM patients. The same mutation in the family may express different phenotypes (HCM, DCM, and RCM). Large ischemic scars have been reported by pathologists and are nowadays easily detectable in vivo by cardiac magnetic resonance with gadolinium. The ischemic arrhythmic substrate enhances the risk of sudden death.

More than 65 years have elapsed since the first pathological report at autopsy of hypertrophic cardiomyopathy by Donald Teare at the St. George’s Hospital in London in eight cases [1]. The gross phenotype was indeed characterized by the asymmetric hypertrophy of the ventricular septum (Figure 1a), with a bizarre disorganization of the myocardial fibers under a light microscope (“myocardial disarray”) (Figure 1b).

Table 1 reports the clinico-pathologic data of the eight cases [1]. Six were male, two were female. The age ranged from 14 to 45 years old, with a mean age of 27.5. Symptoms were reported in four cases: palpitations with atrial fibrillation in three (one with embolic stroke) and syncope in one. Fatal outcome with sudden death occurred in seven cases and postoperative exitus in one.

At the end of the article, he added a “post-scriptum”: “On 13 December 1956, K. C., aged 16, a brother of female case No. 5, collapsed and died while riding his bicycle. No previous medical history was available. At post mortem, he was found to be a well-nourished and well-developed young boy, whose heart was virtually identical in appearance with that of his sister, showing a localized hypertrophy affecting the anterior wall and interventricular septum. By coincidence, on the day of his death, another younger sister attended the outpatient department of Hammersmith hospital and was found to have identical signs. This family will be the subject of another paper”.

In the same year, at the NIH in Bethesda, USA, Eugene Braunwald (Figure 2) and Andrew Glenn Morrow (Figure 3) clinically faced an equally morbid entity with systolic murmur with subaortic stenosis and subvalvular gradient at cardiac catheterization [2]. Eugene Braunwald wrote: “In early 1958, shortly after my appointment as head of the cardiac catheterization laboratory of the National Heart Institute (now the NHLBI) in Bethesda, Maryland, I faced a challenging problem. We had studied a 27-year-old man with progressively severe precordial pain and exertional dyspnoea; a heart murmur had been detected some years earlier. He exhibited a left ventricular (LV) lift and a Grade 4/6 systolic murmur at the apex and along the left sternal border. His ECG showed LV hypertrophy and the chest X-ray suggested LV enlargement. Catheterization showed a subaortic gradient of 74 mmHg” (Figure 3a) [2].

When Andrew Glenn Morrow (Figure 3b), Chief of Cardiac Surgery, planned to resect an alleged discrete fibrous subaortic stenosis [3], he observed only hypertrophy and performed a myectomy of the ventricular septum to relieve the obstruction (Figure 4).

Morrow felt that he could readily resect the obstructing membrane of the patient with a cardiopulmonary bypass and with the heart in arrest. However, he wrote: “we were shocked that at operation no intraventricular obstruction was found” [3].

It is interesting that the terminology employed by Braunwald and Morrow in 1959 was “*functional*” stenosis of the left ventricle outflow [3]. A year later, they definitively labeled the diseases as “*Idiopathic hypertrophic subaortic stenosis*” [4]. The clinical phenotype was asymmetric septal hypertrophy, as observed by Teare at autopsy, with systolic murmur, familial occurrence, and sudden arrhythmic death [2].

In 1957, Lord R. Brock, a cardiac surgeon at the Guy’s Hospital in London, during an operation in extracorporeal circulation and open-heart surgery of alleged aortic valve stenosis, found neither valvular stenosis nor “discrete” subaortic stenosis [5].

The first patient of Braunwald and Morrow, Mr. Brady, later underwent successful cardiac transplantation because of the end-stage disease and is now healthy (Figure 5) [6].

In 1980, Prof. John F. Goodwin (Figure 6) coordinated an international committee [7] of the World Health Organization (WHO), which put forward the first definition and classification of heart muscle disorders (cardiomyopathies) (Figure 7).

“Cardiomyopathies”, including the hypertrophic one, were defined as heart muscle diseases of unknown cause, whereas heart muscle diseases of known cause or associated with disorders of other organs were called “specific heart muscle diseases”. Disorders of the myocardium, caused by systemic or pulmonary hypertension, coronary artery disease, valvular heart disease, and congenital cardiac anomalies, were ruled out [7].

In 1995, the WHO classification was revisited with the addition of two new cardiomyopathies, namely arrhythmogenic and restrictive [8]. By addressing the classification of heart muscle diseases, the name hypertrophic cardiomyopathy was definitively employed [8].

Paradoxically, the same Dr. Morrow was found to be affected by hypertrophic cardiomyopathy. His four-generations genealogical tree showed several members affected with a dominant transmission, with seven cases dying suddenly (Figure 8). He also died suddenly in 1982.

Although a familial occurrence was noticed early in Teare’s report as suspected genetically determined cardiomyopathy, the first disease-related gene was discovered in 1989–1990 by the Seidman group in Boston, consisting of the missense mutation of the β-cardiac myosin heavy chain in chromosome 14 [9,10]. The molecular investigation was made in the same original Teare family (Figure 9) [11,12].

Thus, 23 years elapsed from Teare’s gross discovery to molecular genetic discoveries of the disease. Thus, the same family was the source for both the gross discovery and the genetic explanation of the disease.

After the first genetic discoveries, more than 20 HCM-causing genes have been detected, mainly encoding contractile or structural-regulatory proteins of the sarcomere, myosin chains and myosin-binding protein C included (Figure 10). Nowadays, with a worldwide prevalence of 1:500, HCM is considered the most common genetically determined cardiac disease.

Interestingly enough, some genes responsible for HCM, like the ones coding cardiac troponin, have also been found in primary restrictive cardiomyopathy (RCM) [14,15], a rare cardiac disease characterized by small heart, preserved systolic function, severe diastolic impairment with atrial enlargement, myocardial disarray, and no significant hypertrophy, with a heart failure so severe as to require transplantation [16]. Thus, both HCM and RCM are now considered “sarcomere” diseases and share causative genes coding sarcomeric proteins. Similar gene mutations have also been reported in DCM and non-compaction CM. Moreover, the same mutation in a family may express different cardiomyopathies, and genetic defects have been detected in some DNA non-coding regions of patients with HCM [17].

Diagnostic work-up in HCM has greatly increased during the last 20 years, from abnormal electrocardiographic and hemodynamic findings on M-mode to 2D echocardiography (Figure 11). Since the early 2000s, contrast-enhanced cardiac magnetic resonance (CMR) with gadolinium has emerged as a powerful, non-invasive imaging technique of HCM, allowing the accurate evaluation of hypertrophy, the mitral valve, papillary muscle abnormalities, and ischemic myocardial scarring. The extent of late gadolinium enhancement with CMR is one of the novel markers for the risk stratification of sudden cardiac death in HCM [18].

The progress of management strategies and therapeutic measures have shown that it is now possible to achieve significantly prolonged survival and decrease HCM-related mortality from 6% to <0.5%/year [19].

Sudden cardiac death (SCD), albeit relatively uncommon, is the most fearful event of HCM, particularly in the young. Screening programs for competitive athletes, introduced in Italy since 1982, have proved their efficacy in reducing SCD, mainly thanks to the early diagnosis of HCM to deny sport eligibility [20].

Septal reduction therapies, i.e., surgical myectomy (classical or “modified” Morrow procedure) or percutaneous alcohol septal ablation, proved effective in reducing the gradient, symptoms, and number of patients evolving to progressive heart failure and death. Furthermore, the novel class of specific “myosin inhibitors” seems to be a promising pharmacological option in obstructive forms [21,22]. Heart transplant is the extreme option for end-stage heart failure in patients with nonobstructive HCM who are suffering from an advanced disease and are refractory to pharmacologic management. End-stage heart failure, in the absence of outflow obstruction, is emerging as the predominant risk of death in patients with HCM, since the latter may develop in DCM.

Currently, HCM can be considered a well-known and highly treatable disease, with low morbidity and mortality. Patients with HCM can now aspire to an acceptable quality of life and an almost normal lifespan. However, treatment is still mostly symptomatic, and the management of pediatric HCM is challenging [23].

The prevention of arrhythmic SCD, thanks to the implantable cardioverter defibrillator, has changed the mortality occurrence of the disease.

## 1. The Pathology Spectrum of Hypertrophic Cardiomyopathy

Teare was attracted by the possibility of HCM as a benign tumor. He wrote “Since the term rhabdomyoma is now firmly associated with nodular glycogenic tumours of the heart, it is simpler to refer to the eight tumours under discussion as hamartomata, though they may in fact lay greater claim to being benign tumours of striped muscle than those of presumed glycogenic origin” [1].

**Figure 9:**
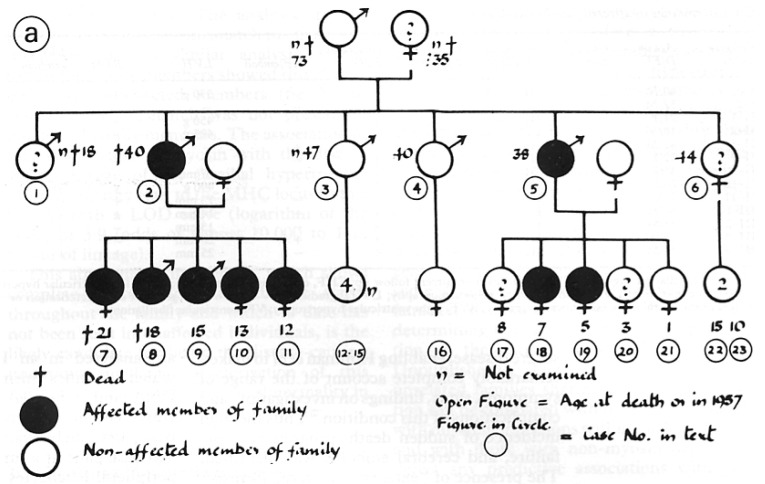
Familial genealogic tree of a young patient who died suddenly in the original series Teare published in 1960 [11].

In 1960, the genealogic tree of a family (three generations) with four sudden death cases, in keeping with a dominant pattern of genetic transmission, was eventually published (Figure 9) [11].

Since the original description by Teare in 1958 [1], a lot of progress in HCM pathology knowledge has been made.

Asymmetric hypertrophy is far more often located in the septal and anterior left ventricle, followed by the posterior ventricular septum, lateral free wall, posterior ventricular wall, and the apex.

**Figure 12:**
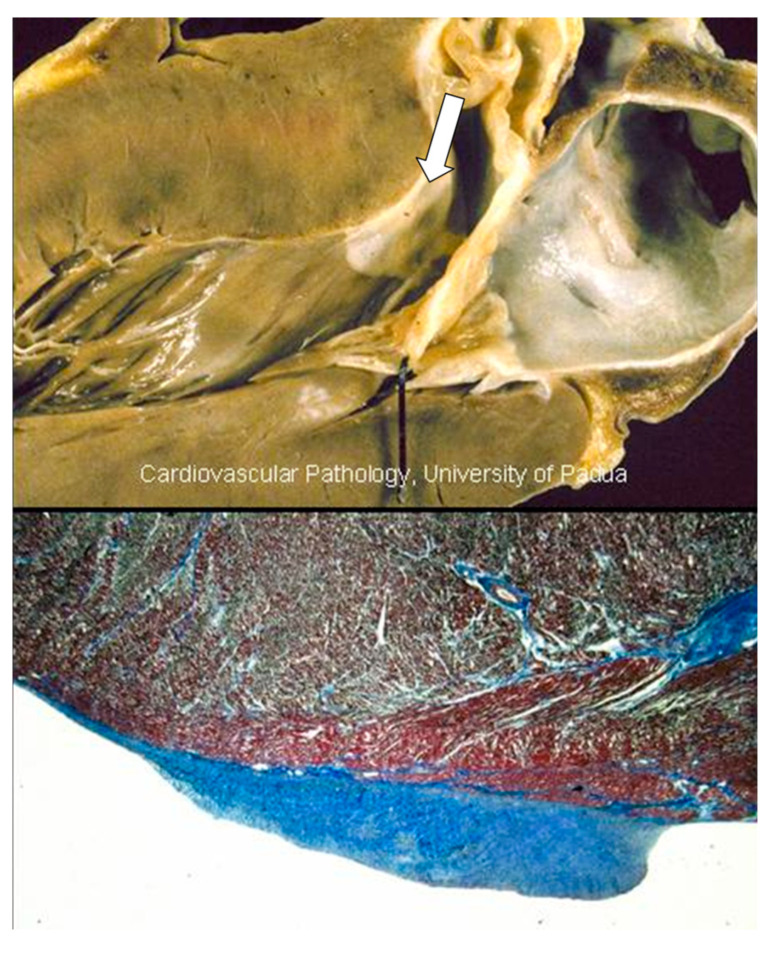
Subaortic endocardial fibrotic plaque (arrow) due to friction between the mitral anterior leaflet and the asymmetric septal hypertrophy.

**Figure 13:**
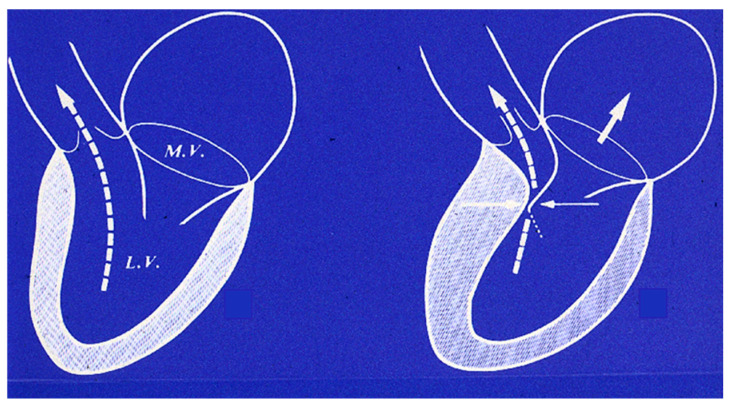
Mechanism accounting for mitral regurgitation and the dilatation of the left atrium in hypertrophic cardiomyopathy. LA = left atrium; LV = left ventricle; and MV = mitral valve.

**Figure 14:**
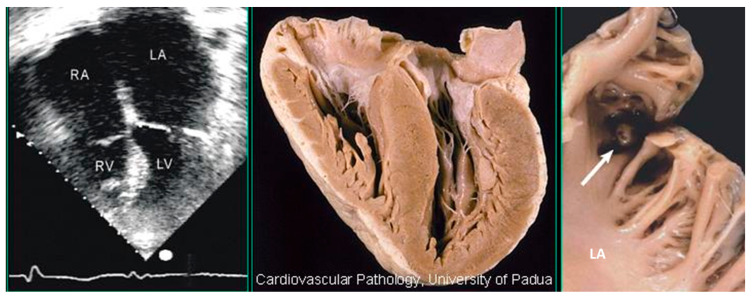
Thrombosis in the left atrial appendage (arrow) of a patient with hypertrophic cardiomyopathy, dilated left atrium, and atrial fibrillation who underwent cardiac transplantation. LA = left atrium; LV = left ventricle; RA = right atrium; and RV = right ventricle.

**Figure 15:**
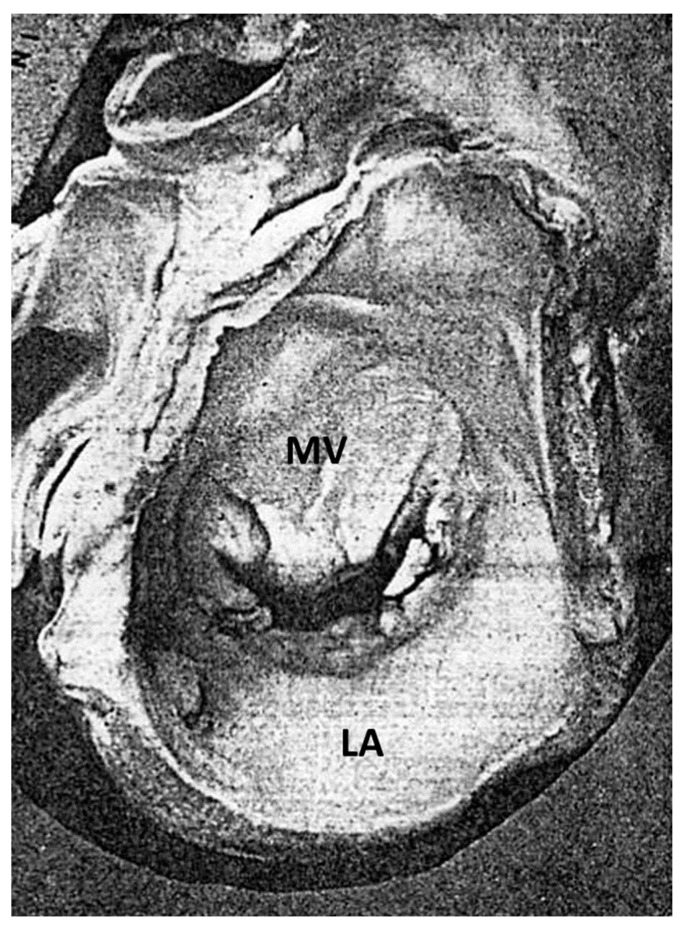
A figure from Teare’s original paper [1] representing a cardiac specimen with a dilated left atrium. LA = left atrium; MV = mitral valve.

A peculiar fibrous plaque grows up with time on the left-sided endocardium of asymmetric ventricular septal hypertrophy, due to the friction of the anterior leaflet of the mitral valve with the ventricular septum during systole (Figure 12). The phenomenon accounts for mild to moderate mitral incompetence, which, together with the diastolic restrictivity by myocardial disarray, entails left atrial dilatation (Figure 13), with the onset of atrial fibrillation and left atrial appendage thrombosis (Figure 14), increasing the risk of embolism and stroke. A dilated left atrium in HCM was also reported in a picture from the original paper by Teare [1] (Figure 15).

**Figure 16:**
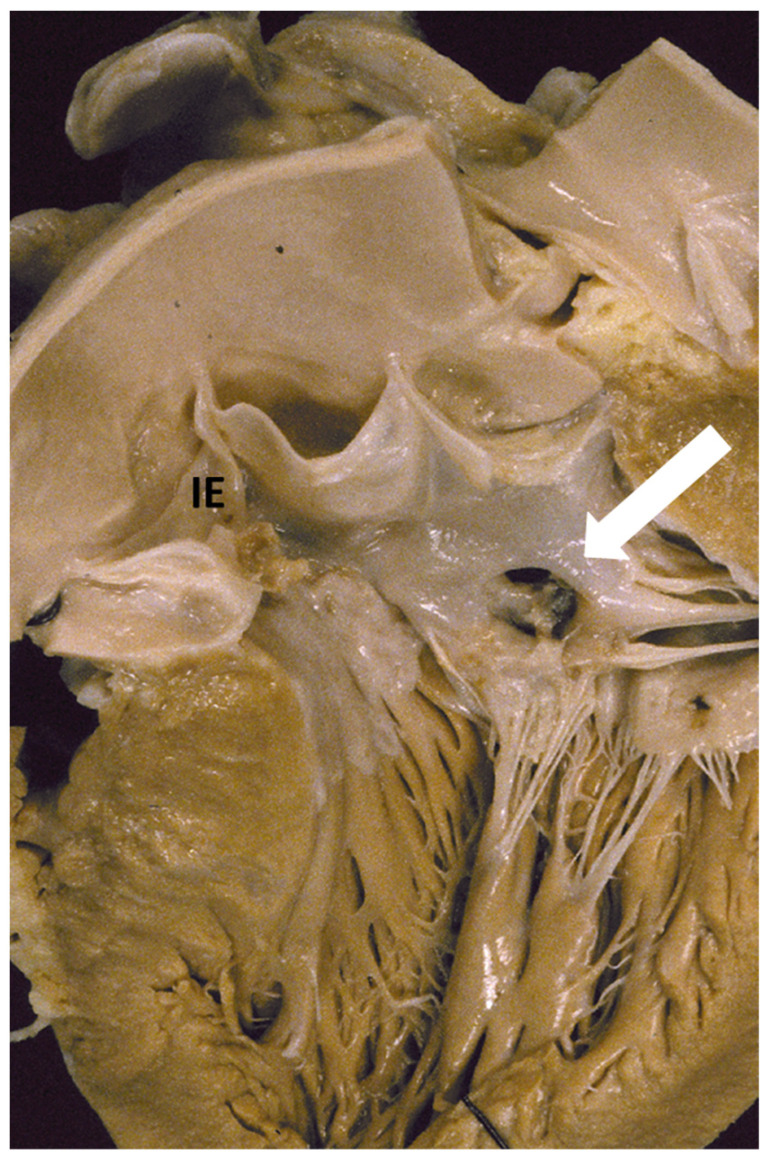
Infective endocarditis (IE), developed on subaortic septal plaque, in a patient with hypertrophic cardiomyopathy. The vegetations conveyed the infection to the anterior leaflet mitral valve through a “kissing” mechanism, with perforation (arrow) and aneurysm. The infection also involved the aortic valve.

The fibrous plaque growing over the left-sided endocardium of the asymmetric septal hypertrophy may be a substrate facilitating infective endocarditis onset in case of bacteremia. Transfer of the infection may occur from the septum to the anterior mitral leaflet (“kissing lesion”) and the aortic cusps, with the onset of vegetations, cusp perforation, and aneurysm (Figure 16).

**Figure 17:**
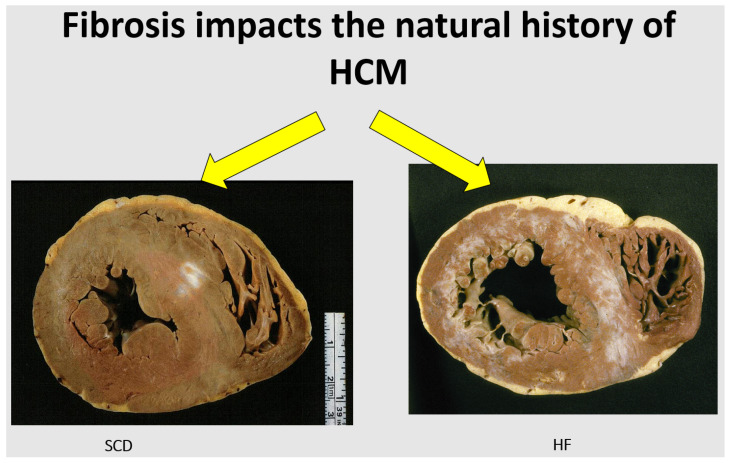
Infarct-like scars of the ventricular septum and free wall of the left ventricle in end-stage hypertrophic cardiomyopathy.

Another complication, occurring in the natural history of hypertrophic cardiomyopathy, is myocardial scarring [24,25]. It may involve both the interventricular septum and the free wall (Figure 17). This is the so-called end stage of hypertrophic cardiomyopathy, accounting for severe congestive heart failure and requiring cardiac transplantation as an extreme therapeutic option.

**Figure 18:**
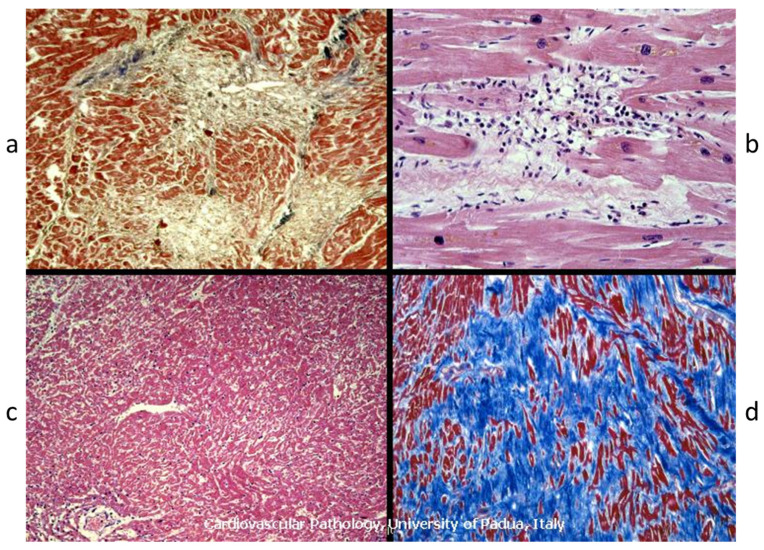
Acute ischemic myocardial damage in hypertrophic cardiomyopathy at various histologic stages. Hematoxylin and eosin stains at high (**b**) and low (**a**,**c**,**d**) magnifications.

Obstructive disease of small arteries has been called into question, despite fibrotic areas having been observed even in the absence of small arteries disease. At histology, the early lesions are clearly ischemic (Figure 18), and large scars show the shape of a previous myocardial infarct (Figure 17).

Multiple mechanisms of myocardial ischemia and necrosis have been postulated, from an increase in oxygen demand (LV hypertrophy, diastolic impairment, LV outflow tract obstruction, and arrhythmias) to decreased myocardial perfusion (small vessel disease and increased coronary vascular resistance). Increased intramural myocardial resistance during diastole, when myocardial perfusion occurs, seems to be the most reliable mechanism.

**Figure 19:**
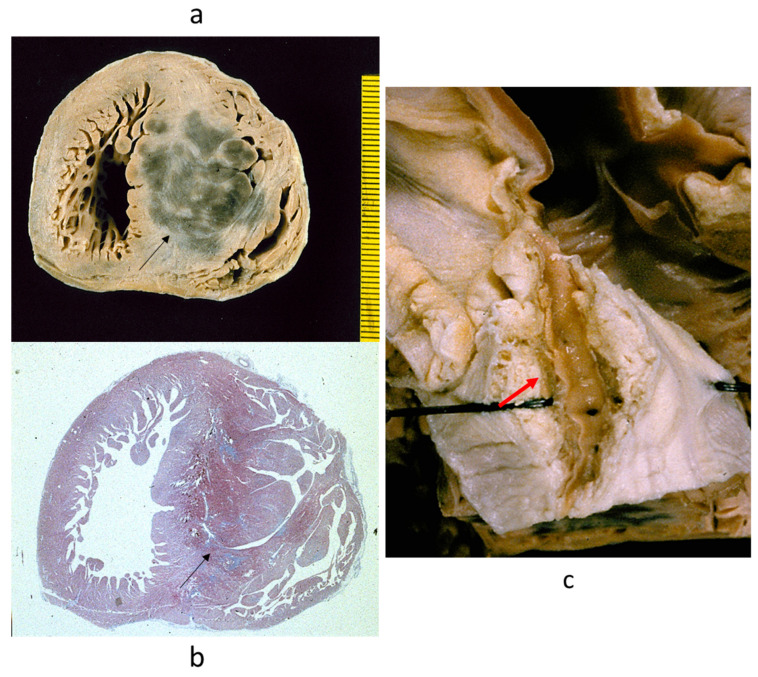
(**a**,**b**) Massive infarction of the ventricular septum. Note the huge asymmetric hypertrophy (arrows) and (**c**) deep, long intramural course of the anterior descending coronary artery (arrow).

Another postulated cause was the intramural course (myocardial bridge) of the descending coronary artery [26], with cases reported with massive acute anteroseptal myocardial infarction and sudden death (Figure 19).

However, large scars have been reported in the ventricular septum, even in the absence of a myocardial bridge and the deep intramyocardial course without myocardial infarction. The question remains intriguing [26].

**Figure 20:**
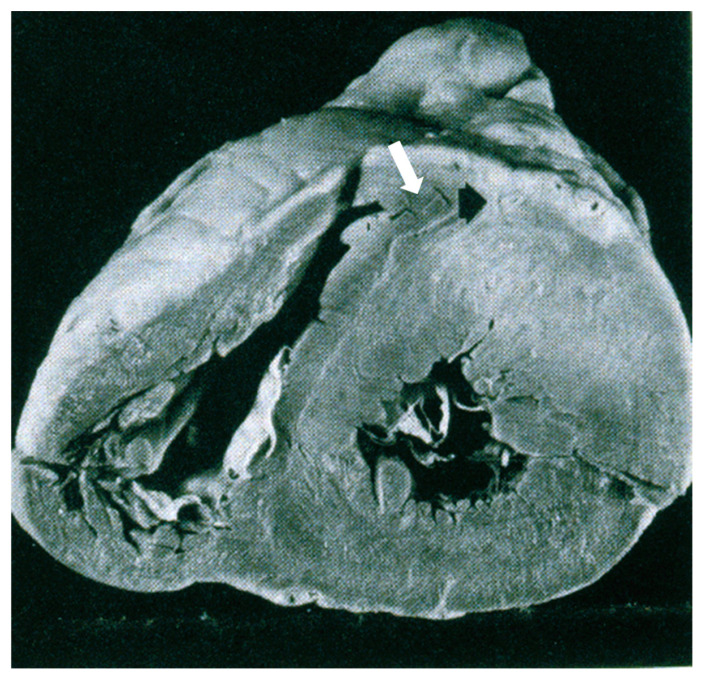
This is the original illustration of the publication of the intramural course of the anterior descending coronary artery (ADCA) as an isolated cause of sudden death. However, it is quite evident that it is a case of hypertrophic cardiomyopathy with anteroseptal asymmetric hypertrophy and intramural course (arrow) of ADCA. Permission was obtained from [27].

The myocardial bridge has even been accused of being an isolated cause of sudden death in the absence of hypertrophic cardiomyopathy. Checking the illustration of the original report at the cross-section of the heart specimen [27], it appears as a clear-cut case of hypertrophic cardiomyopathy with antero-septal asymmetric hypertrophy (Figure 20).

Hypertrophic cardiomyopathy is a cause of SCD [20,24,28]. The prospective investigation in the Veneto Region, Italy showed HCM with a rate of 9% among all SDs in the young.

The incidence of HCM in the USA with SCD among athletes is reportedly much higher than that reported in Italy. The preparticipation screening, obligatory in Italy, entails baseline stress test ecgs and even 2D echo, in addition to anamnesis and physical visits. Thus, they easily identify the affected subjects to deny eligibility [20].

A higher risk of life-threatening arrhythmias occurs when asymmetric hypertrophy is complicated by ischemic fibrosis, wherever it is.

## 2. Restrictive Cardiomyopathy

In 1980, at the time of the first WHO classification of heart muscle diseases [7], Loeffer disease, with endomyocardial eosinophilic inflammation infiltrates, mural thrombosis, and ventricular cavities reduction, was named restrictive cardiomyopathy.

**Figure 21:**
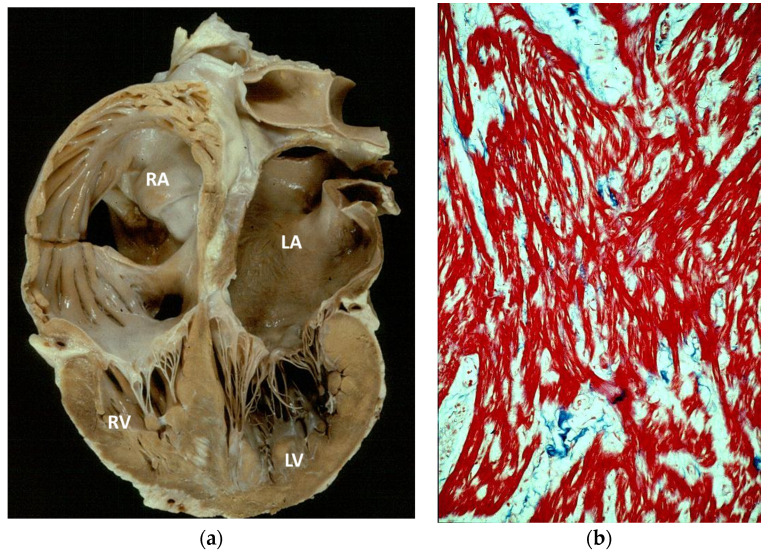
Restrictive cardiomyopathy with normal ventricles, dilated atria (**a**), and ventricular myocardial disarray at histology (**b**). Azan-Mallory stain at high magnification. LA = left atrium; LV = left ventricle; RA = right atrium; RV = right ventricle.

**Figure 22:**
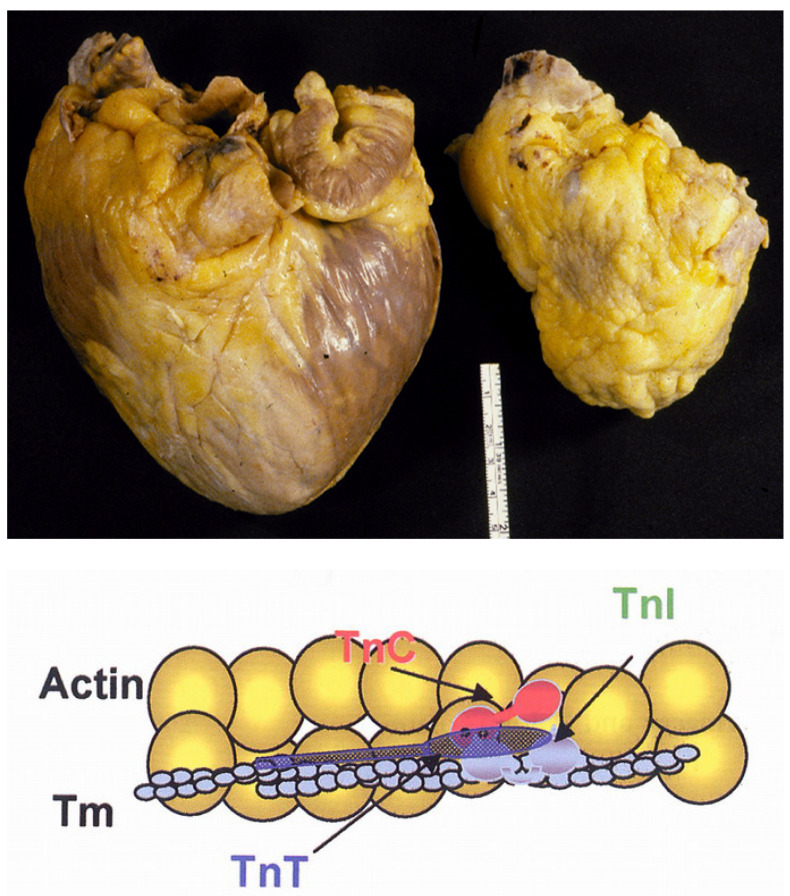
The paradox of a small heart in restrictive cardiomyopathy (on the right) requiring transplantation, compared to the “cor bovinum” of dilated cardiomyopathy (on the left). A genetic mutation of troponin I (low) was found [14]. Tm= tropomyosin; TnC = troponin C; TnI = troponin I; TnT = troponin T.

Later, in the 1980s–1990s, a new form, with an intact endocardium, was reported as primary heart muscle disease, mostly impairing the diastole. Both the atria appeared dilated like “appendices” of the ventricles (Figure 21a). The histology of the ventricular myocardium showed myocardial disarray in the absence of ventricular hypertrophy (Figure 21b). It was proved to be a genetically determined sarcomeric myocardial disease, due to troponin I mutations (Figure 22) [14]. We wonder whether it should be considered a “hypertrophic cardiomyopathy” without hypertrophy, with severe diastolic dysfunction, accounting for atrial fibrillation and left atrial appendage dilatation at risk of thrombosis, embolism, and stroke, as well as congestive heart failure requiring cardiac transplantation.

## 3. Conclusions

Enormous progress has been made in the last decades in HCM knowledge; however, many issues remain to be solved, especially in cell and molecular biology for a better specific treatment.

The creation of animal models of HCM for research [29], which have already helped shed light on pathophysiology mechanisms for the development of targeted treatments, is desirable.

Moreover, gene therapy [30], with the replacement of the morbid gene with a healthy one, may be an achievable dream, defeating a transmissible, genetically determined disease like HCM with its disappearance forever.

## Figures and Tables

**Figure 1:**
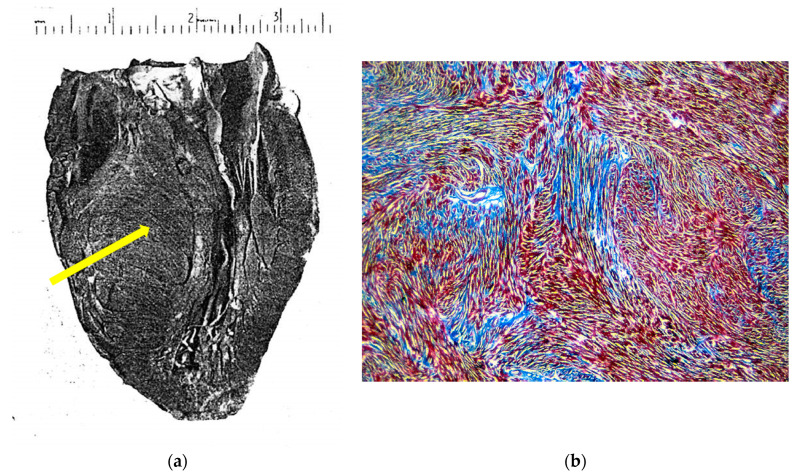
(**a**) The original picture of a cardiac specimen from the Donald Teare paper [1]. Coincidentally, the cut was performed along the long axis of the left ventricle, similar to the current 2D echo tomographic section, depicting the septal asymmetric hypertrophy (arrow). (**b**) Myocardial disarray under a light microscope, mimicking a hamartoma. Azan-Mallory stain, low magnification.

**Figure 2:**
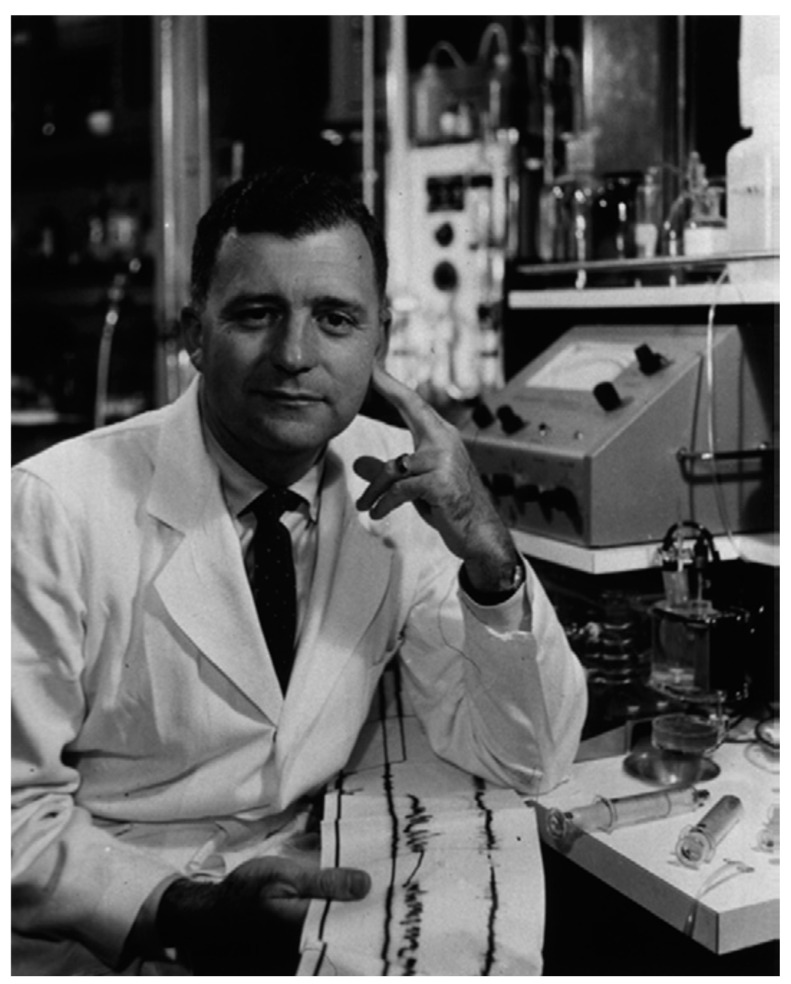
Picture of the young Eugene Braunwald in the 1960s at the National Institutes of Health (NIH), chief of hemodynamics.

**Figure 3:**
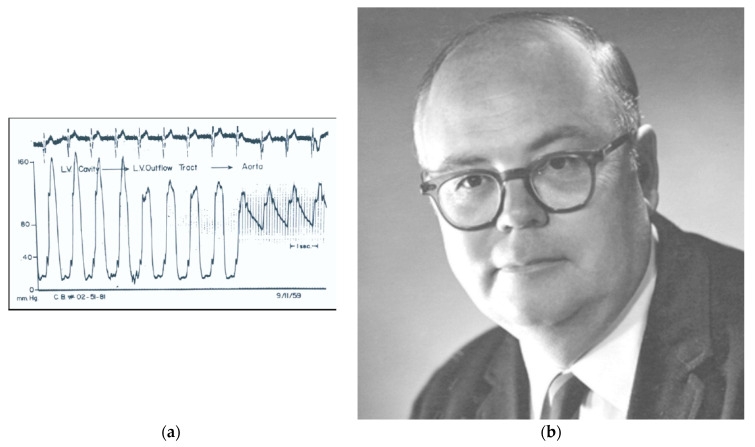
(**a**) Mr. Brady’s continuous pullback pressure tracing, recorded as the catheter was withdrawn from the LV cavity through the outflow tract across the aortic valve and into the aorta. Reproduced with permission from [2]. (**b**) Andrew Glenn Morrow’s picture, chief of Cardiac Surgery at the NIH in the 1960s. He wrote the paper reporting the first patient with HCM and subaortic gradient [3].

**Figure 4:**
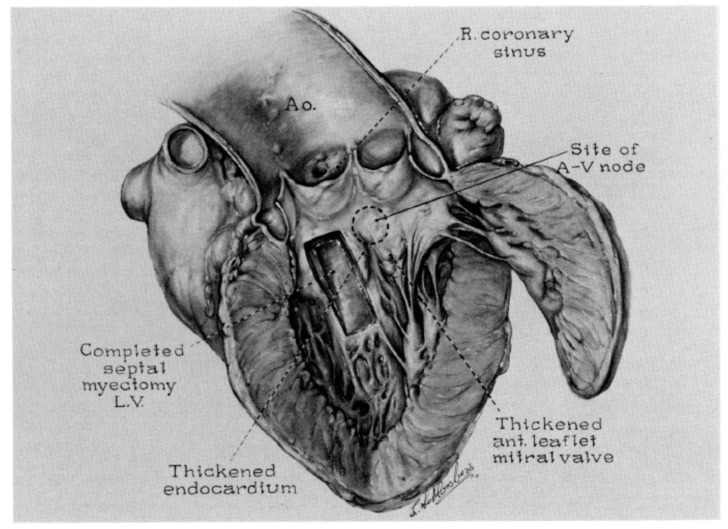
The surgical myectomy technique, invented by Morrow.

**Figure 5:**
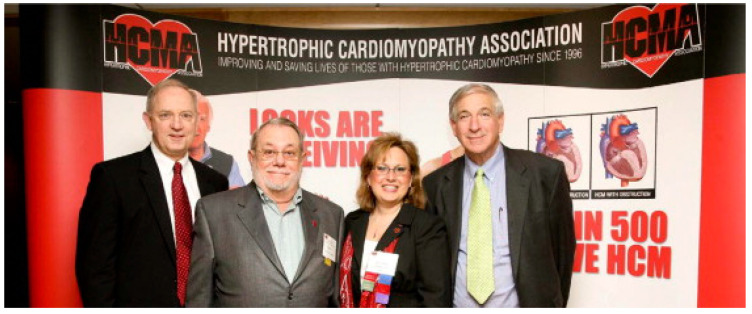
A recent picture of Mr. Brady, the first patient in the USA diagnosed as affected by idiopathic hypertrophic subaortic stenosis (with Dr. Bonow (**left**), Mrs. Salberg (**middle**), and Dr. Maron (**right**)) [6].

**Figure 6:**
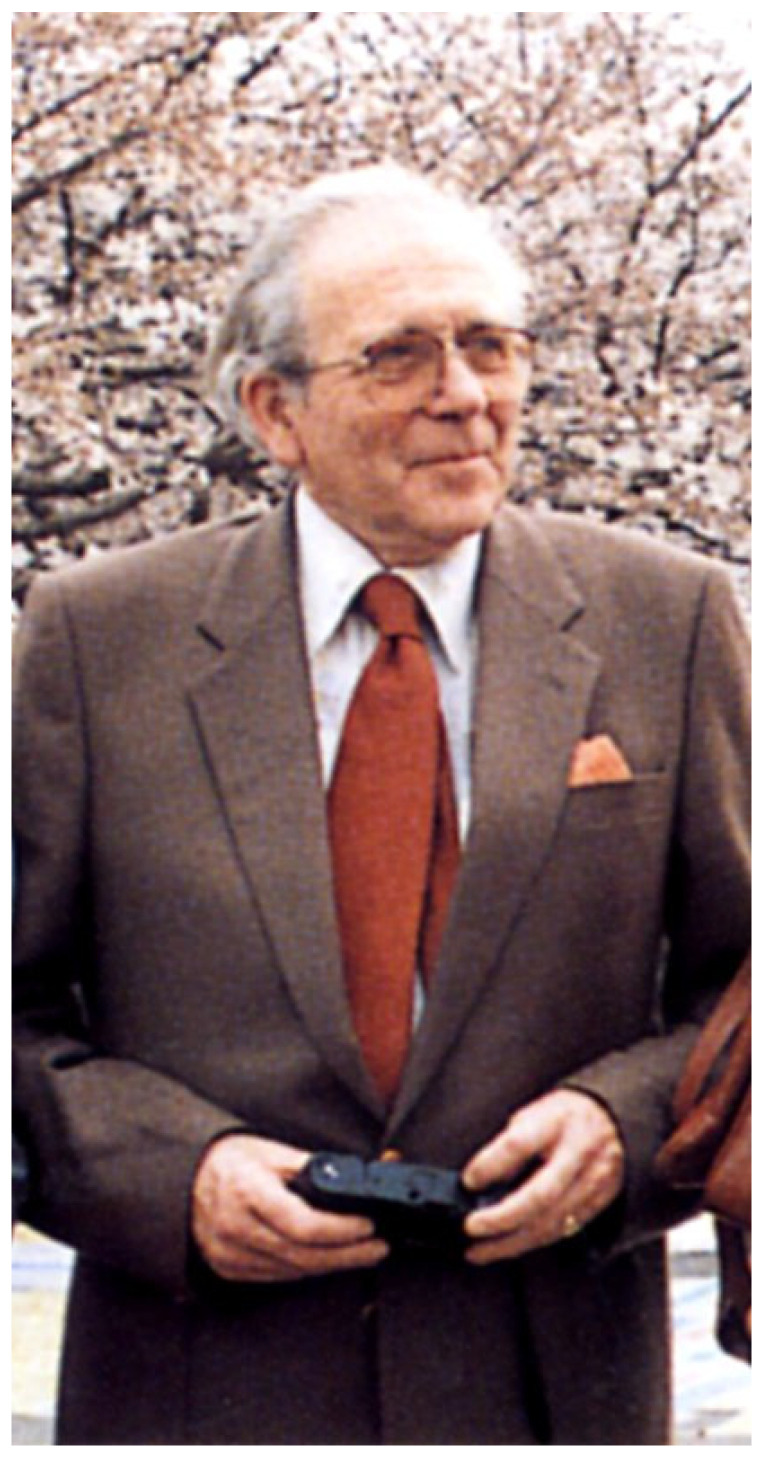
John F. Goodwin, Chief of Cardiology at the Hammersmith Hospital in London, who in 1980 published the first WHO classification of primary heart muscle diseases, introducing the term cardiomyopathy [7].

**Figure 7:**
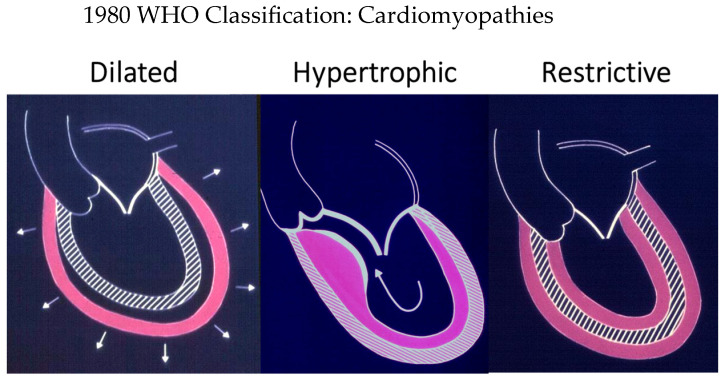
The three cardiomyopathies of the 1980 WHO classification: dilated, hypertrophic, and restrictive, the latter referring to Loeffer eosinophilic endomyocardial disease.

**Figure 8:**
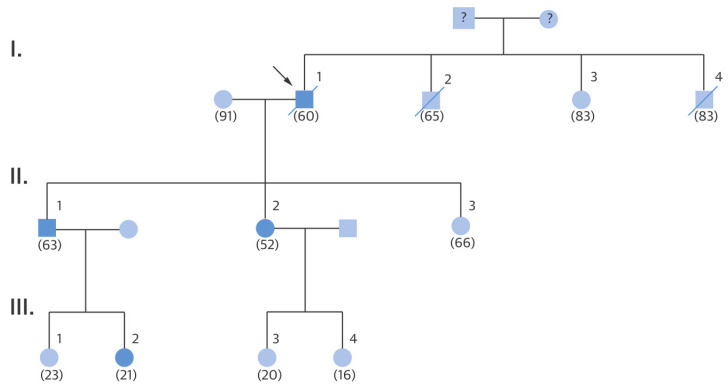
Morrow Family Pedigree. Family genealogic tree of Dr. Morrow, who was affected by hypertrophic cardiomyopathy and died suddenly in 1982. Squares indicate men; circles indicate women; the arrow indicates the proband (Dr. Morrow, I.1.); solid symbols indicate hypertrophic cardiomyopathy; and the slash indicates deceased.

**Figure 10:**
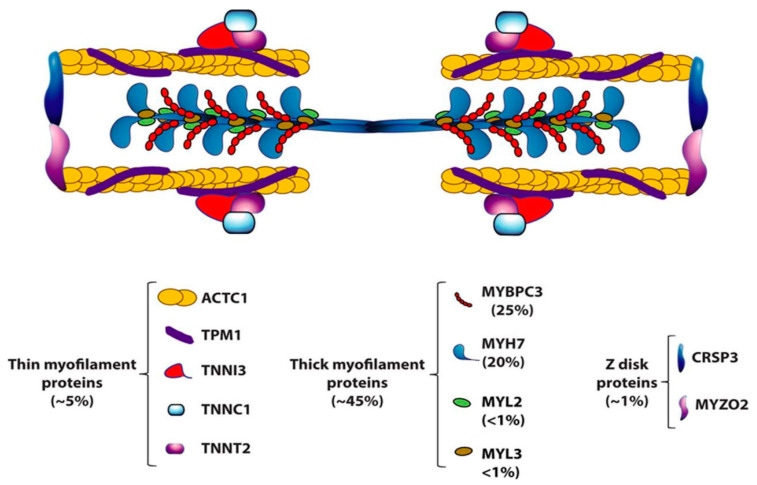
Sarcomeric proteins, carried by missense mutations of causative genes in HCM (from [13]), with permission.

**Figure 11:**
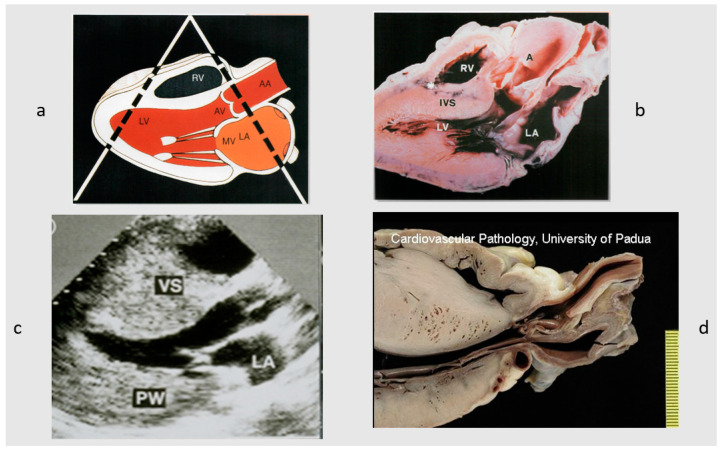
Image of 2D echocardiography, the revolutionary non-invasive diagnostic technique for an easy visualization of asymmetric hypertrophy. (**a**) Schematic long-axis tomographic view at 2D cardiac echo. (**b**) The equivalent in a normal heart. (**c**) The 2D echo in hypertrophic cardiomyopathy. (**d**) Cardiac specimen of (**c**), with septal asymmetric hypertrophy. A = aorta; AA = ascending aorta; AV = aortic valve; LA = left atrium; LV = left ventricle; PW = posterior wall; RV = right ventricle; and VS = ventricular septum.

**Table 1:** Original demographic and pathology findings of the original Tears’ paper [1]. Another parents was added as postscriptum.

8 cases (6 M, 2 F) Age: 14–45 yrs, mean 27.5 Symptoms in 4 − Palpitations with AF: 3 (embolic stroke in 1) − Syncope (“blackout”): 1 Exitus − Sudden death: 7 − Post-operative fatal outcome: 1 Pathology: − Asymmetrical hypertrophy of the interventricular septum and of the anterior wall of the left ventricle − Myocyte disarray and ischemic fibrosis
